# Large-scale screening for severe acute respiratory coronavirus virus 2 (SARS-CoV-2) among healthcare workers: Prevalence and risk factors for asymptomatic and pauci-symptomatic carriers, with emphasis on the use of personal protective equipment (PPE)

**DOI:** 10.1017/ice.2021.68

**Published:** 2021-04-24

**Authors:** Sandra Rajme-López, María F. González-Lara, Edgar Ortiz-Brizuela, Carla M. Román-Montes, Janet Santiago-Cruz, Miguel Ángel Mendoza-Rojas, Steven Méndez-Ramos, Karla M. Tamez-Torres, Esteban Pérez-García, Bernardo Alfonso Martínez-Guerra, Luz Elena Cervantes-Villar, Pilar Ramos-Cervantes, Violeta Ibarra-González, David Kershenobich-Stalnikowitz, José Sifuentes-Osornio, Guillermo M. Ruíz-Palacios, Alfredo Ponce-de-León

**Affiliations:** 1 Infectious Diseases Department, Instituto Nacional de Ciencias Médicas y Nutrición Salvador Zubirán, Mexico City, Mexico; 2 Clinical Microbiology Laboratory, Instituto Nacional de Ciencias Médicas y Nutrición Salvador Zubirán, Mexico City, Mexico; 3 Virology Laboratory, Instituto Nacional de Ciencias Médicas y Nutrición Salvador Zubirán, Mexico City, Mexico; 4 General Direction, Instituto Nacional de Ciencias Médicas y Nutrición Salvador Zubirán, Mexico City, Mexico; 5 Department of Medicine, Instituto Nacional de Ciencias Médicas y NutriciónSalvador Zubirán, Mexico City, Mexico,

## Abstract

Healthcare workers (HCWs) not fulfilling the coronavirus disease 2019 (COVID-19) case definition underwent severe acute respiratory coronavirus virus 2 (SARS-CoV-2) screening. Risk of exposure, adherence to personal protective equipment (PPE), and symptoms were assessed. In total, 2,000 HCWs were screened: 5.5% were positive for SARS-CoV-2 by polymerase chain reaction (PCR). There were no differences in PPE use between SARS-CoV-2–positive and –negative HCWs (adherence, >90%). Nursing and kitchen staff were independently associated with positive SARS-CoV-2 results.

Healthcare workers (HCWs), especially in resource-limited countries, are a vulnerable population for severe acute respiratory coronavirus virus 2 (SARS-CoV-2) infection. Government data show that >20% of coronavirus disease 2019 (COVID-19) cases in Mexico have occurred among HCWs, with >1,000 HCWs deaths.^1^ SARS-CoV-2 PCR-positive cases not fulfilling the CDC case definition are considered pauci-symptomatic, but definition of this category is imprecise. Asymptomatic transmission of SARS-CoV-2 is a major reason for its rapid spread.^2^ Limiting quarantine, reducing spread, and protecting the healthcare work force^3^ are benefits of testing asymptomatic HCWs. Previous studies to identify risk factors in healthcare settings are inconclusive.^4^ We sought to determine the prevalence of asymptomatic and pauci-symptomatic SARS-COV-2 carriers among HCWs and to identify potential risk factors.

## Methods

This prevalence study was conducted at a COVID-19 center in Mexico City, between April 28 and July 8, 2020. HCWs aged ≥65 years or with comorbidities remained at home according to the national policy. HCWs without COVID-19 suspicion who voluntarily signed informed consent were included. Study participants with symptoms not fulfilling the COVID-19 case definition were considered pauci-symptomatic, and those without symptoms were considered asymptomatic. This study was approved by the ethics and research committees.

An outdoor, ventilated, HCW COVID-19 testing facility was implemented. Participants answered an electronic questionnaire based on the World Health Organization (WHO) tool for assessing exposure risk and use of personal protective equipment (PPE).^5^ Nasopharyngeal (NP) swabs were obtained by trained personnel. Nucleic acid amplification (NAAT) testing and real-time reverse transcription-polymerase chain reaction were performed as described elsewhere.^6^ Results were communicated via e-mail within 48 hours. Study participants who were PCR positive for SARS-CoV-2 received instructions about isolation, symptoms, and warning signs and were asked to return for another NP swab 7 days later. For PCR-positive HCWs, we assessed symptom development, duration, need for hospitalization, and incidence of domiciliary COVID-19 cases.

We classified HCWs in 7 categories and work areas in 10 categories (Table 1). We performed subgroup analyses among HCWs with the highest SARS-CoV-2–positive prevalence to compare key characteristics. Descriptive analyses of demographics, clinical data, workplace characteristics, and PPE use were performed using, standard deviation, median, and interquartile range, as appropriate. Comparative analyses were performed using the χ^2^ test and the Fisher exact test. Univariate analyses of factors associated with being PCR positive for SARS-CoV-2 were performed, followed by multivariate logistic regression including variables with a *P* value ≤.15 or biological plausibility.

**Table 1.** Comparison of clinical characteristics, PPE use and AGPs between SARS-CoV-2 PCR+ and PCR– HCWs

	All 2000 (%)	PCR+ 111 (5.5)	PCR– 1889 (94.5)	*P* Value
**I. GENERAL CHARACTERSITICS**				
Sex, male (n, %)	851 (42.5)	50 (45)	801 (42)	.50
Age (median, IQR)	34 (28-46)	34 (26-43)	34 (28-46)	.13
Weight, (median, IQR)	69 (60-80)	70 (60-82)	69 (60-80)	.50
Body mass index (median, IQR)	26 (23-29)	26 (23-29)	26 (23-29)	.23
**Comorbidities**				.52
Overweight/obesity	1229 (61.5)	76 (68.5)	1153 (61)	.12
Hypertension	80 (4)	2 (1.8)	78 (4.1)	.22
Diabetes mellitus	29 (1.5)	2 (1.8)	27 (1.4)	.75
Ischemic heart disease	5 (0.3)	1 (0.9)	4 (0.2)	.16
Chronic lung disease^a^	45 (2.3)	3 (2.7)	42 (2.2)	.74
Current smoker	341 (17.1)	15 (13.5)	326 (17.3)	.31
**Type of work**				
Medical	562 (28.1)	24 (21.6)	538 (28.5)	.12
Nursing	551 (27.6)	44 (39.6)	507 (26.8)	.003
Administrative	298 (14.9)	16 (14.4)	282 (14.9)	.88
Paramedical	196 (9.8)	5 (4.5)	191 (10.1)	.05
Cleaning	172 (8.6)	11 (9.9)	161 (8.5)	.61
Kitchen	73 (3.6)	8 (7.3)	65 (3.5)	.04
Other	148 (7.4)	3 (2.7)	145 (7.7)	.05
**Workplace**				
COVID-19 critical areas	612 (30.5)	38 (34.2)	574 (30.4)	.39
COVID-19 general ward	477 (23.8)	34 (30.6)	443 (23.4)	.09
Administrative building or office	238 (11.8)	10 (9.0)	228 (12.1)	.33
Non–COVID-19 outpatient clinic	224 (11.2)	7 (6.3)	217 (11.4)	.09
Laboratory	162 (8.1)	6 (5.5)	156 (8.3)	.28
Radiology	55 (2.8)	1 (0.9)	54 (2.9)	.22
Hospital staff triage	47 (2.4)	2 (1.8)	45 (2.4)	0.69
Staff kitchen	43 (2.2)	6 (5.4)	37 (1.9)	.015
Radio-oncology unit	31 (1.6)	3 (2.7)	28 (1.5)	.31
Other	111 (5.6)	4 (3.6)	107 (5.7)	.36
Previous exposure to a COVID-19 case	1226 (61.3)	81 (73)	1145 (60.6)	.009
**Site of first exposure to a COVID-19 case**				.47
Hospital	1156 (94.3)	75 (92.6)	1081 (94.4)	
Outside of hospital	70 (5.7)	6 (7.4)	64 (5.6)	
Face masking at work place	1669 (83.5)	98 (88.3)	1571 (83.2)	.16
Direct care of COVID-19 patients	974 (48.7)	63 (56.8)	911 (48.2)	.08
AGPs	682 (34.1)	47 (42.3)	635 (33.6)	.059
Contact with the environment of COVID-19 patients	1120 (56)	72 (64.9)	1048 (55.5)	.053
Symptoms 7 days before testing	1067 (53.4)	80 (72.1)	987 (52.3)	.000
**II. PPE USE AMONG HCWs DIRECTLY CARING FOR COVID-19 PATIENTS**				
**Face mask**				
Any face mask	907 (93.1)	57 (90.5)	850 (93.3)	.39
Surgical face mask	672 (69)	43 (68.3)	629 (69.1)	.89
N95 face mask	658 (67.6)	44 (69.8)	614 (67.4)	.67
Gloves	872 (89.5)	56 (88.9)	816 (89.6)	.86
Goggles	856 (87.9)	55 (87.3)	801 (87.9)	.88
Face shield	455 (46.7)	31 (49.2)	424 (46.5)	.68
**Gown**				
Any gown	819 (84.1)	55 (87.3)	764 (83.9)	.47
Disposable gown	551 (56.6)	43 (68.3)	508 (55.8)	.053
Reusable gown	526 (54)	34 (54)	492 (54)	.99
Hair cap	511 (52.5)	36 (57.1)	475 (52.1)	.44
Shoe covers	462 (47.4)	33 (52.4)	429 (47.1)	.42
Correct removal of PPE	910 (93.4)	57 (90.5)	853 (93.6)	.33
Hand hygiene after contact with a COVID-19 patient	961 (98.7)	61 (96.8)	900 (98.8)	.19
**III. TYPE AND PPE USE DURING AEROSOL-GENERATING PROCEDURES**				
**Type of AGP**				
Tracheal intubation	318 (46.6)	19 (40.4)	299 (47.1)	.38
Nebulizer treatment	83 (12.2)	5 (10.6)	78 (12.3)	.73
Open airway suctioning	374 (55.1)	22 (46.8)	352 (55.7)	.24
Collection of sputum	299 (44)	18 (38.3)	281 (44.5)	.41
Tracheostomy/bronchoscopy	64 (9.4)	4 (8.5)	60 (9.5)	0.82
Collection of nasophrayngeal swab	157 (23.1)	7 (14.9)	150 (23.7)	0.17
CPR	58 (8.5)	1 (2.1)	57 (9.0)	0.10
N95 face mask	638 (93.6)	42 (89.4)	596 (93.9)	.23
Gloves	671 (98.4)	45 (95.7)	626 (98.6)	.14
Goggles	649 (95.2)	43 (91.5)	606 (95.4)	.22
Face shield	345 (50.6)	25 (53.2)	320 (50.4)	.71
Gown	662 (97.1)	45 (95.7)	617 (97.2)	.58
Correct removal of PPE	653 (95.8)	47 (100)	606 (95.4)	.13
Hand hygiene after AGPs	677 (99.3)	47 (100)	630 (99.2)	.54

Note. PPE, personal protective equipment; AGP, aerosol-generating procedure; PCR+, positive polymerase chain reaction assay for SARS-CoV-2; PCR−, negative PCR assay for SARS-CoV-2; HCW, healthcare worker; CPR, cardiopulmonary resuscitation.

a Asthma, chronic obstructive pulmonary disease (COPD), or interstitial lung disease (ILD).

## Results

Overall 2,000 HCWs were screened: 111 (5.5%) were PCR positive (nursing 8%, medical 4%, paramedical 3%, administrative 5%, cleaning 6%, kitchen 11% and other staff 2%); 933 HCWs (46.7%) were asymptomatic; and 1,067 HCWs (53.4%) were pauci-symptomatic. Age, comorbidities, and other characteristics are described in Table 1. Most participants were medical and nursing staff (28.1% and 27.6% respectively); 48.7% (974) were directly involved in COVID-19 patient care. The most common workplace areas were critical care units (30.5%) and general wards (23.8%). HCWs who were PCR positive for SARS-CoV-2 were distributed as follows: 44 (39.6%) of 111 nurses, 24 (21.6%) of 111 medical staff (residents and attendings), 16 (14.4%) of 111 administrative assistants, 11 (9.9%) of 111 cleaning staff, 8 (8.3%) of 111 kitchen staff, 5 (9.9%) of 111 paramedic staff, and 3 (2.7%) of 111 other HCWs.

Among the 111 HCWs positive for SARS-CoV-2, 31 (21.9%) were asymptomatic; the rest were pauci-symptomatic. Common symptoms were odynophagia (41.4%), headache (38.7%), rhinorrhea (31.5%), cough (17.1%), arthralgia or myalgia (13.5%), red eyes (12.6%), olfactory and/or taste disorders (11.7%), diarrhea (10.8%), fever (7.2%), dyspnea (3.6%), and skin lesions (0.9%). A second NP swab was available for 104 (93.7%), of which 50 (48%) were PCR positive for SARS-CoV-2. On follow-up, 17 of these 111 HCWs (15.3%) remained asymptomatic and 14 (14.6%) developed new symptoms. Among pauci-symptomatic participants, 32 (40%) developed mild COVID-19. One HCW required admission and was discharged uneventfully. Also, 27 study participants (24.6%) reported ≥1 household contact diagnosed with COVID-19 within 7 days after their positive result.

The SARS-CoV-2 PCR cycle threshold (Ct) value was not associated with pauci-symptomatic state (OR, 0.83; 95% CI, 0.36–1.93), symptom development (OR, 0.97; 95% CI, 0.42–2.27), or positive household contact (OR, 1.41; 95% CI, 0.58–3.41). We detected no differences in PPE use among HCWs caring directly for COVID-19 patients between SARS-CoV-2–positive and –negative HCWs (Table 1).

On univariate analysis, nursing, kitchen staff, exposure to a COVID-19 case, and working in a COVID-19 environment were associated with being PCR positive for SARS-CoV-2. On multivariate analysis, nursing and kitchen staff remained independently associated (Table 2).

**Table 2.** Univariate and Multivariate Analyses of Characteristics Associated With Being SARS-Cov-2 PCR+

Characteristic	Univariate		Multivariate	
	OR (95% CI)	*P* Value	OR (95% CI)	*P* Value
**Comorbidity**				
Overweight/obesity	1.39 (0.92–2.1)	.12		
Hypertension	0.43 (0.10–1.76)	.24		
Diabetes mellitus	1.26 (0.30–5.39)	.75		
Ischemic heart disease	4.28 (0.48–38.7)	.20		
Chronic lung disease^a^	1.22 (0.37–4.00)	.33		
**Current smoker**	0.75 (0.43–1.31)	.31		
**Type of work**				
Medical	0.69 (0.43–1.10)	.12		
Nursing	1.79 (1.21–2.65)	.004	1.66 (1.01–2.73)	.04
Administrative	0.96 (0.56–1.65)	.88		
Paramedical	0.42 (0.17–1.04)	.06		
Cleaning	1.18 (0.62–2.25)	.51		
Kitchen	2.18 (1.02–4.66)	.045		
**Workplace**				
COVID-19 critical areas	1.19 (0.80–1.79)	.39	1.06 (0.50–2.24)	.88
COVID-19 general ward	1.44 (0.95–2.19)	.09	1.48 (0.79–2.77)	.22
Administrative building/office	0.72 (0.37–1.40)	.33		
Non-COVID-19 outpatient visits	0.52 (0.24–1.13)	.10	0.75 (0.32–1.74)	.49
Laboratory	0.63 (0.27–1.47)	.29		
Radiology	0.31 0.04–2.25)	.25		
Hospital staff triage	0.75 (0.18–3.14)	.70		
Staff kitchen	2.86 (1.18–6.92)	.02	3.95 (1.53–10.2)	.005
Radio-oncology unit	1.84 (0.55–6.17)	.32		
Other	0.63 (0.23–1.72)	.36		.88
Exposure to a COVID-19 case	1.75 (1.14–2.69)	.01		
Face masking at workplace	1.52 (0.85–2.76)	.16		
Directly taking care of COVID-19 patients	1.41 (0.96–2.17)	.08	0.98 (0.50–1.92)	.94
In charge of AGP	1.45 (0.98–2.14)	0.06	1.15 (0.61–2.19)	0.65
Contact with the environment of COVID-19 patients	1.48 (0.99–2.2)	0.05		

Note. PCR+, positive polymerase chain reaction assay for SARS-CoV-2; CI, confidence interval; AGP, aerosol-generating procedure.

a Asthma, chronic obstructive pulmonary disease (COPD), or interstitial lung disease (ILD).

Compared with medical staff, nurses positive for SARS-CoV-2 more frequently worked in critical areas (61.4% vs 33.3%; *P* = .007) and more frequently performed aerosol-generating procedures (AGPs; 72.7% vs 54.2%; *P* = .04). Subgroup analyses among positive cases are described in Supplementary Table 3 (online).

## Discussion

We found a prevalence of 5.5% SARS-CoV-2 infection among asymptomatic and pauci-symptomatic HCWs, consistent with previous reports (3%–5%).^7,8^ Nursing staff comprised most cases; paramedical and kitchen staff were also frequently positive for SARS-CoV-2. HCWs caring directly for COVID-19 patients were <50% of our study participants, which may be explained because they were less likely to be screened due to work overload.

The ideal PPE for frontline HCWs remains controversial.^9^ We assessed PPE use among HCWs caring directly for COVID-19 patients. Adherence to PPE use and hand hygiene was high (>90%). We found no differences between surgical or N95 masks nor between goggles or face shields, among HCWs positive or negative for SARS-CoV-2. Hair and shoe covers were used by <50% and were not associated with being PCR negative for SARS-CoV-2.

Nursing staff caring directly for COVID-19 patients were positive for SARS-CoV-2 more frequently, especially when working in critical care areas and performing AGPs. The time inside patient rooms was not assessed. Long periods in nonventilated rooms might influence the positivity rate. Medical staff at the outpatient clinic and paramedical not involved in COVID-19 areas were positive for SARS-CoV-2 more frequently. The occurrence of a COVID-19 case among kitchen staff motivated exposed HCWs to undergo SARS-CoV-2 testing, explaining the elevated prevalence in this subgroup. Authorities were informed, and working conditions were revised (ie, ventilation, temperature, and masking). No additional work-related factors were identified.

Pauci-symptomatic HCWs accounted for 53.4% of the study population and 72% of the HCWs positive for SARS-CoV-2. Odynophagia and headache were the most frequent symptoms, but they were attributed to stress and PPE use. Symptom minimization and work overload may explain why some HCWs did not seek timely attention.

Low Ct values have been associated with severe disease, risk of intubation and mortality.^10^ The relation between low Ct value and presymptomatic or pauci-symptomatic disease or with the transmissibility of SARS-CoV-2 have yet to be defined. In our study, low Ct values were not associated with being positive for SARS-CoV-2 nor with household cases.

This study had several limitations. Self-reporting may have led to recall and reporting bias regarding the use and adherence to PPE, as well as symptoms. Community exposure to SARS-CoV-2 (public transportation, social gatherings, etc) was not evaluated. Night-shift HCWs and those caring directly for COVID-19 patients participated less frequently. Contact tracing was self-reported, so the possibility of underreporting cannot be excluded. Finally, we did not perform viral genome-sequencing analysis, making it impossible to determine in-hospital transmission.

This study also has several strengths. The assessment of PPE use was thorough, and we considered a variety of HCW roles. Also, the identification of HCWs positive for SARS-CoV-2 led to their isolation, which may have diminished the spread of SARS-CoV-2. Pauci-symptomatic disease has been poorly characterized. Including this group of HCWs may lead to a broader definition of COVID-19. Finally, placing the screening area near the main entrance led to a greater interest in participating.

In conclusion, the prevalence of SARS-CoV-2 among asymptomatic and pauci-symptomatic HCWs in a COVID-19 center in Mexico City was 5.5%. Nurses in critical areas represented the majority of PCR-positive tests. High adherence to PPE recommendations was observed, suggesting community transmission as the most likely source of infection.

## Supplementary material

For supplementary material accompanying this paper visit http://dx.doi.org/10.1017/ice.2021.68. click here to view supplementary material
