# Supplementary material for: Large-scale screening for severe acute respiratory coronavirus virus 2 (SARS-CoV-2) among healthcare workers: Prevalence and risk factors for asymptomatic and pauci-symptomatic carriers, with emphasis on the use of personal protective equipment (PPE)
Source: Infect Control Hosp Epidemiol. 2021 Apr 24:1–5. doi: 10.1017/ice.2021.68 (PMC8111176; doi:10.1017/ice.2021.68)
Supplement: Supplementary file 1 [file S0899823X21000684sup001.docx]

Supplementary material

Table 3. Relevant characteristics among groups with high prevalence of asymtpomatic/pauci-symptomatic carrier state

|  | **All PCR +**  **111 (%)** | **Nursing staff**  **PCR +**  **44 (60.3%)** | **Medical staff**  **PCR +**  **24 (32.9%)** | **Other staff**  **PCR +**  **43 (38.7%)** | p |
| --- | --- | --- | --- | --- | --- |
| **Any face mask** | 98 (88.3) | 41 (93.2) | 22 (91.7) | 35 (81.4) | 0.19 |
| **Direct care of COVID-19 patients** | 63 (56.8) | 39 (88.6) | 18 (75) | 6 (13.9) | 0.000 |
| **Workplace**  COVID-19 critical areas  COVID-19 general ward  Non COVID-19 outpatient clinic  Laboratory  Hospital staff triage  Staff kitchen  Other | 38 (34.3)  34 (30.6)  7 (6.3)  6 (5.4)  2 (1.8)  6 (5.4)  4 (3.6) | 27 (61.4)  15 (34.1)  0  0  0  0  0 | 8 (33.3)  9 (37.5)  3 (12.5)  1 (4.2)  2 (8.3)  0  1 (4.2) | 3 (6.9)  10 (23.2)  4 (9.3)  5 (11.6)  0  6 (13.9)  3 (6.9) | 0.000  0.39  0.04  0.05  0.03  0.007  0.22 |
| **Aerosol-generating procedures** | 47 (42.3) | 32 (72.7) | 13 (54.2) | 2 (4.7) | 0.000 |

Figure 1. Distribution of cases based on current personal address


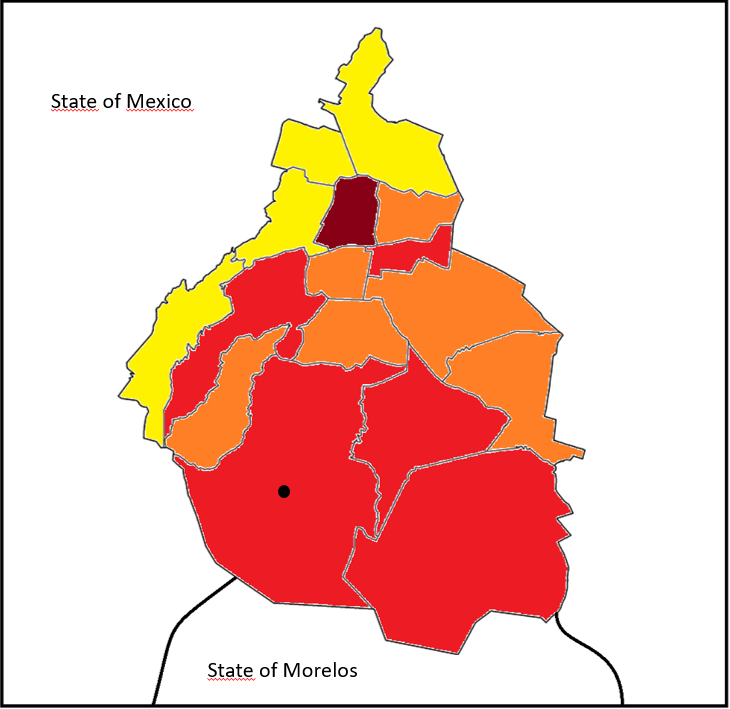


Mexico City is divided into 16 counties. Percentage of PCR positivity among HCWs from our study is marked on the map as follows: dark red >10%, red >5-10%, orange 1-5%, yellow <1%. Two hundred healtchare workers lived outside Mexico City. Sate of Mexico PCR positivity percentage was 8.3% and State of Morelos 6.3%. This distribution is consistent with Mexico´s City community transmission hot spots during the months of May through mid July^26^.

*Black dot indicates location of our Institution
